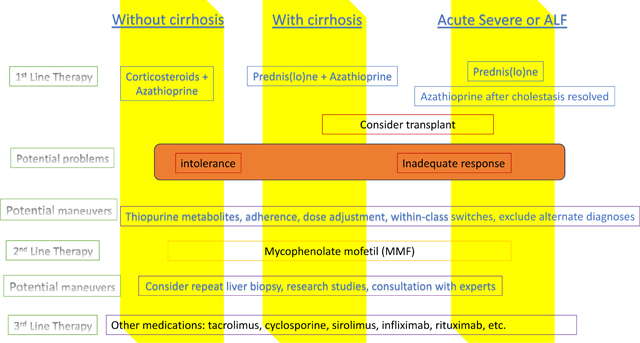# AIH Therapy: Beyond First‑Line

**DOI:** 10.1007/s11901-024-00657-4

**Published:** 2024-03-08

**Authors:** Irina Adao, Arielle Klepper, Michele Tana

**Affiliations:** 1 University of California, San Francisco, 1001 Potrero Avenue, Building 5, Unit 3D, San Francisco, CA 94110, USA

**Keywords:** Autoimmune hepatitis, Second-line therapy, Third-line therapy, AIH treatment

## Abstract

**Purpose of Review:**

The purpose of the article is to review treatment options for patients with AIH for whom first-line therapy is not successful. We outline recommended approaches for providers and new therapies on the horizon.

**Recent Findings:**

Budesonide, while advantageous in some respects, may not be as effective as predniso(lo)ne. Mycophenolate mofetil is most effective in the setting of azathioprine intolerance and less effective when the response to azathioprine has been inadequate. Infliximab is the biologic agent with the most evidence for use in AIH. Clinical trials studying interleukin 2, regulatory T cells, inhibitors of BAFF signaling, and immunoproteasome inhibitors have been initiated but more research is needed, particularly in Black people, Indigenous people, and People of Color.

**Summary:**

While multiple agents have been reported as second- or third-line therapies, the evidence is limited. Future research will require multicenter collaboration and should explore therapeutics supported by molecular studies.

## Introduction

In this review, we discuss issues that come up during first-line therapy for AIH, review the literature on second- and third-line therapies, and explore molecular mechanisms that could inform future therapeutic approaches.

While second-line therapies form the crux of this review, first-line therapy is hardly straightforward. Corticosteroids are the cornerstone of treatment in AIH, yet the optimal dosing strategy and method of monitoring response (i.e., frequency of laboratory testing) have not been determined. As corticosteroids are tapered, enzymes often flare. This can require adjusting the corticosteroid dose and rethinking the maintenance regimen. Repeated flares are thought to promote fibrosis progression. Furthermore, fibrosis stage and side effects experienced by the patient may lead providers to switch the type of corticosteroid used. In addition, corticosteroids are notoriously disliked by patients, who are clamoring for steroid-free treatments.

Thiopurine methyltransferase (TPMT) enzyme activity level should be checked near the time of diagnosis. A low TPMT activity level either eliminates the possibility of azathioprine use or warrants very close monitoring of the patient for the development of cytopenias while on the drug. Unfortunately, given the dearth of effective options for AIH treatment, providers and patients find themselves in a difficult situation when the TPMT activity level is even slightly low. Azathioprine has long been considered our first choice for maintenance therapy, but this approach is not entirely evidence-based. To address this, the multicenter CAMARO trial just closed in the Netherlands, looking at azathioprine vs. MMF for maintenance therapy in treatment-naive patients with AIH [1]. Intolerance of azathioprine also occurs, in which case substituting the prodrug 6-MP can lessen GI side effects. Alternatively, the addition of allopurinol can favorably alter the metabolism of azathioprine to reduce toxic metabolites and increase therapeutic metabolites [2]. Monitoring thiopurine metabolite levels is perhaps an underutilized tool in the management of patients with AIH, as these results can help differentiate nonadherence from thiopurine toxicity and guide dose adjustment.

Interestingly, treatment response after just 8 weeks predicts long-term outcome like achievement of complete remission. Budesonide, while initially found to be as effective as prednisone in achieving remission [3], may be inferior based on a retrospective study from Spain [4]. Remission rate with a second-line agent is higher if the reason for switching from the first agent was intolerance rather than inadequate response.

Groups like the American Association for the Study of Liver Diseases and European Association for the Study of Liver have issued guidance, but quality indicators in AIH management are lacking. Changes to guidance in recent years include the recommendation against using budesonide in acute severe presentations, the optionality of liver biopsy prior to withdrawal of therapy, and the added complexity of patients who have both AIH and metabolic dysfunction-associated steatotic liver disease. A recent paper found that PNPLA variants are associated with outcomes in AIH [5]. While patients and providers may wish to avoid invasive liver biopsies, many experts emphasize the crucial information this procedure sometimes yields, namely, the development of a variant syndrome (overlap) with PBC and the degree of histologic inflammation (sometimes much more or much less than expected based on blood test results alone).

Given the complexity of even first-line therapy, and the vulnerable populations disproportionately affected by AIH, the field needs to consider **system-level changes** to improve outcomes in AIH. Some examples are efforts to promote earlier diagnosis/screening, continuing medical education of primary-care providers and gastroenterologists, ancillary staff to help patients navigate their care, organized patient education (classes, workshops, and resources available in patients’ primary language), the establishment and measurement of quality indicators, and the expansion of clinical trials, with a goal to increase the number of available medications targeting pathways implicated in AIH pathophysiology. While many health disparities have been documented in AIH, research is needed to understand root causes and then intervene on relevant factors.

Patients and providers frequently encounter issues with first-line therapy. Though some of the maneuvers mentioned above can help exhaust first-line approaches, this article will review several alternative therapies beyond first-line.

### Moving Beyond First‑Line

The indications for moving to the next line of therapy can be broadly divided into two categories: (1) those who are intolerant to the previous therapy due to adverse effects of the medication and (2) those who did not adequately respond to treatment. The latter category can be further subdivided into treatment failure, which is defined as worsening of laboratory and histological findings, or incomplete response, which is defined as an improvement of laboratory and histological levels but not to the level of remission [6].

Of note, biochemical remission is defined as normalization of AST, ALT, and IgG on blood tests. Histologic remission refers to absent to minimal inflammation on microscopic exam of a pathologic liver specimen [6–10]. However, there remains no official consensus for treatment regimens beyond first-line therapy.

The literature surrounding 2nd- and 3rd-line therapy mostly comprised retrospective cohort studies, case series, and case reports, but the body of evidence is continuing to grow (Table 1). The primary outcome for most of these studies is complete/biochemical remission. The TAILOR study is a multicenter randomized controlled trial currently underway in the Netherlands, comparing mycophenolate and tacrolimus as second-line agents for patients not achieving a complete remission with first-line therapy [11].

### Mycophenolate Mofetil

Mycophenolate mofetil (MMF) is the most studied and one of the most commonly used 2nd-line medications. MMF is a prodrug that ultimately disrupts the de-novo synthesis of purines [12]. In one real-world clinical study, MMF had a remission rate of 75% as a first-line therapy for autoimmune hepatitis alongside prednisone [13]. As second-line therapy, its response rates vary from 32 to 82%, depending on the indication for switching therapies [14]. MMF was found to be more effective as a second-line drug if the indication for its use was intolerance of side effects from standard therapy [12, 14–16]. MMF has also been shown to have lower efficacy as a second-line therapy in AIH patients with cirrhosis compared to those without cirrhosis [12, 17]. This may be due to treatment-resistant disease rather than low drug efficacy, as there seemed to be no difference in cirrhotic vs non-cirrhotic patients’ response rate with MMF as a first-line therapy [12, 13]. MMF has been used as monotherapy with high efficacy in those intolerant to corticosteroids [15], which may warrant future studies on MMF as part of steroid-free regimens. MMF is generally well-tolerated in adults and children, with pediatric response rates varying from 36 to 55% when used as second-line therapy [18, 19]. However, MMF’s use is limited by its teratogenicity. Other known adverse effects of MMF also include hematologic suppression, gastrointestinal side effects, and headache [20••].

### Calcineurin Inhibitors

Calcineurin inhibitors, tacrolimus (TAC) and cyclosporine (CsA), are widely used in organ transplantation, and they have also been used off-label as alternate agents for AIH. CNIs treat AIH by diminishing the immune response through inhibition of T lymphocyte proliferation [21]. TAC as second- or third-line therapy has been shown to achieve biochemical remission rates ranging from 53 to 78% [21, 22], while CsA achieved remission rates of approximately 59% [21, 23]. In a retrospective study comparing TAC and MMF as second-line therapy, both had excellent response rates, though there was no significant difference between the two [20••]. Unlike MMF, CNIs are not teratogenic and are safe to use in patients who wish to get pregnant. However, one drawback with CNIs is that they must be monitored with trough levels. This translates to frequent lab draws for patients, which may not be desirable. There is no universally agreed target trough level for TAC, but one paper reports that a trough lower than 5 ng/mL is still efficacious and can minimize long-term toxicities [22]. Recommended CsA trough levels are 150–200 ng/mL and can be tapered once in remission [24]. In terms of safety, CNIs are generally well tolerated. However, TAC has been discontinued for neurological side effects, ototoxicity, hypertension, and renal failure [20, 22] while CsA has caused renal failure, gingival hypertrophy, and hypertension [21, 24, 25]. Both CNIs and MMF have been associated with increased risk of malignancy [26].

### Sirolimus

Sirolimus inhibits mammalian target of rapamycin (mTOR) and is commonly used to avoid rejection in organ transplants. Data regarding its use in AIH, however, is extremely limited and lacking in recent literature. One longitudinal follow-up study showed that 4 out of 5 patients responded to sirolimus with 2 patients achieving full remission [27]. There has also been success in adding sirolimus when treating posttransplant AIH in pediatric patients [28]. Complications of sirolimus include infection and elevation of cholesterol and triglyceride levels [27, 28].

### Methotrexate

Methotrexate (MTX) is another medication used to treat AIH in a few case reports and case series. Though AASLD does not include MTX in their 2019 practice guidelines, EASL does mention MTX as an anecdotally used drug in their 2015 guidelines. A 2018 case series demonstrated complete biochemical remission in 54.5% of their 11 patients [29]. Methotrexate was also successful in inducing biochemical remission and becoming steroid-free in 2 pediatric patients [30]. However, serious adverse effects, such as hepatotoxicity, pulmonary fibrosis, and hematologic suppression, and its teratogenicity can limit MTX’s use in AIH [31]. Notably, 2 patients in the case series developed drug-induced liver injury so lab monitoring is necessary if initiating MTX [29].

### Biologic Agents

Biologic agents have also been used in the treatment of AIH, though have not been studied enough to recommend as treatment. Infliximab is a monoclonal antibody against tumornecrosis factor alpha (TNF-α) that is commonly used to treat other autoimmune disorders. It has also been used as rescue therapy for AIH. It had moderate success with concurrent steroid use in a small retrospective cohort study with a complete remission rate of 55% [32]. However, infectious complications of varying severity were seen in the majority of patients, with one patient having to discontinue due to the infection [32]. Furthermore, having cirrhosis can increase the infection risk. Infliximab can also cause hepatotoxicity, and there have been case reports of infliximab-induced AIH. Most resolved with the withdrawal of infliximab, though two patients resulted in a liver transplant [33–35].

Rituximab, a monoclonal antibody against CD20, is another biologic agent that has been used off-label for AIH. In one multicenter, retrospective cohort study, rituximab significantly improved transaminases and IgG levels and was well-tolerated [36]. Interestingly, it also significantly reduced the steroid dose in 62% of patients [36]. BAFF inhibitors are a new option for third-line therapy. They inhibit B cell activating factor (BAFF), which dampen the immune response involved in the pathogenesis of AIH. Currently, there are 2 case reports and a case series that showed improved disease control with adjunctive use of belimumab in patients with refractory AIH [37, 38].

Interestingly, the general trends within the literature show that AIH patients who were intolerant to previous therapy typically respond better to next-line therapy compared to those who were non-responders. We suspect that non-responders likely may have more resistant disease and, thus, have a lower likelihood of responding to any therapy. In comparison, those who were intolerant may only adversely react to that specific drug and can be treated with an alternative regimen instead.

Despite the guidelines issued by different organizations, there remains no conclusive algorithm for drug of choice in the 2nd- and 3rd-line therapy for AIH. More recently, the European Reference Network on Hepatological Diseases and the International Autoimmune Hepatitis Group have suggested a treatment algorithm for 2nd- and 3rd-line therapy options, in which they recommend MMF as second-line treatment but no definitive recommendations on third-line therapy, further highlighting the paucity in current literature [7•]. Though medications such as MMF or CNIs are more frequently used and studied, even data for these therapies are limited to retrospective cohort studies, case series, and case reports. Biologics such as infliximab and rituximab have weak evidence for efficacy in AIH. However, other forms of immunotherapy, such as BAFF inhibitors, are promising and may provide more options in the future. When deciding the next line of therapy, physicians should also factor in the patients’ goals while considering each therapy’s potential side effects, monitoring requirements, and patient comorbidities.

### Molecular Trends From Patient‑Based AIH Studies

**Fig. 1:**
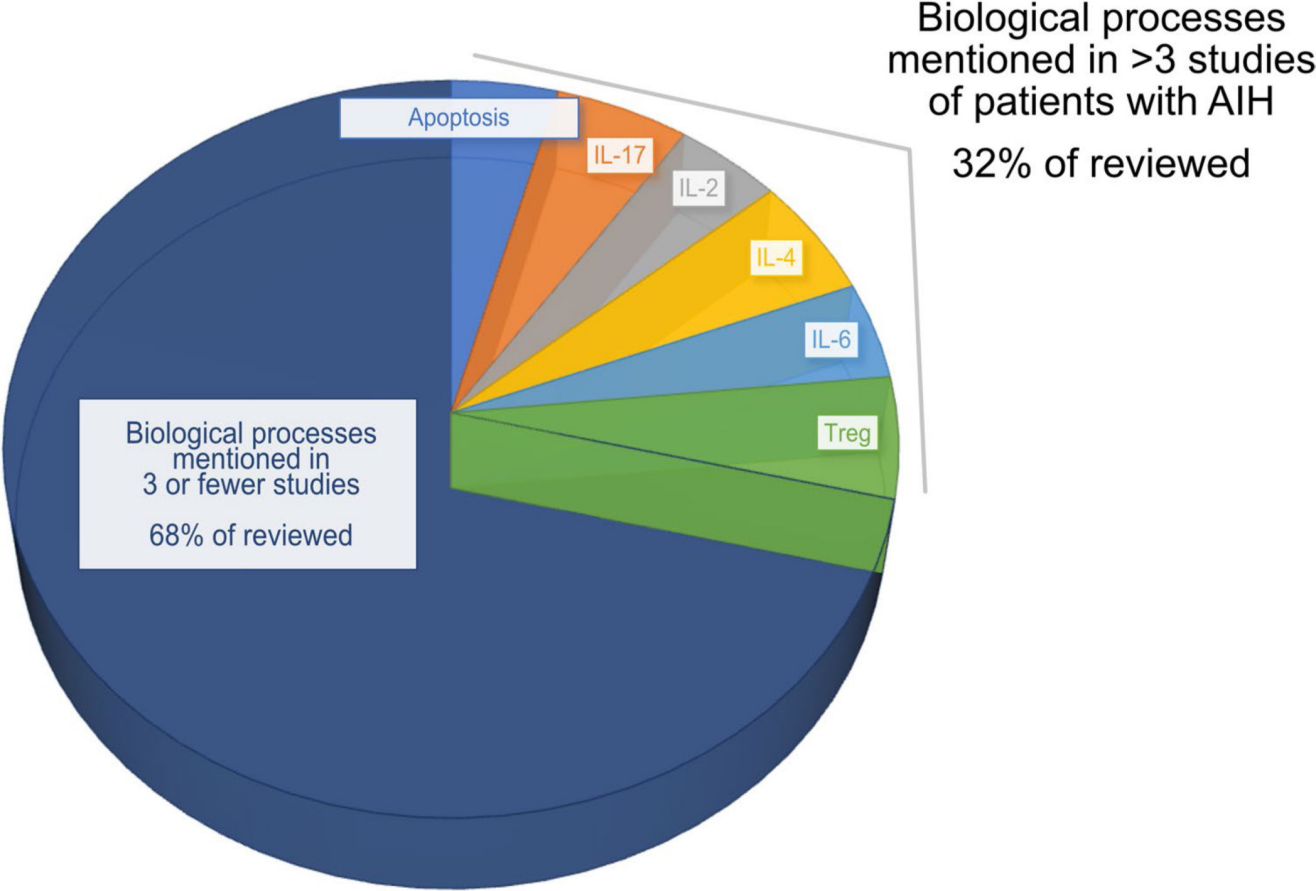
Patient-based studies on AIH. Biologic processes mentioned in >3 studies are shown in the top right of the chart. Processes mentioned in <3 studies are shown in navy blue

Both American and European liver societies have identified that developing new treatments for autoimmune hepatitis is major unmet need in the field [6, 26], and thus, this remains an active area of research. However, we lack a molecular understanding of disease pathogenesis in AIH, which limits our ability to rationally select therapies targeting specific pathways. Leaders in the field have generated a broad compendium of immunomodulatory and emerging therapies on the horizon, which have been comprehensively reviewed elsewhere, such as in the 2019 AASLD guidance on AIH (Table 14) [6]. To complement this work, we used a systematic approach to identify trends in molecular studies on AIH pathogenesis to highlight pathways that are commonly reported as being significant in patients with AIH (Fig. 1). We searched PubMed for articles that shared 3 concepts: autoimmune hepatitis, measurement methods, and substances measured; a list of search terms can be found in Appendix 1. We then screened this list to include studies focused on humans and that tested a hypothesis, thus excluding mouse and in vitro only studies, as well as excluding case reports and descriptive studies. We selected articles that mention potential therapeutic application of their results, and then reviewed the findings from these studies to identify the biological processes that were found to influence AIH pathogenesis or response to treatment.

We identified 2066 studies using our search terms, with 16% of studies meeting our criteria for review. We placed a particular emphasis on more recent studies. Among these studies, there was a large diversity of biological processes identified. However, themes did emerge, as several biological processes were identified in more than 3 studies. These themes represent 1/3 of all studies reviewed (see Fig. 1). The most notable theme was among studies pointing to roles for IL-2 and regulatory T cells (Treg), which express high levels of the IL-2 receptor. These two processes are linked, as IL-2 is required for Treg differentiation outside of the thymus [39]. Additional areas of focus include cytokines with various functions: interleukin 17 (IL-17), a cytokine expressed by activated T cells [40]; interleukin 6 (IL-6), a proinflammatory cytokine that drives activated B cells to differentiate into antibody producing cells [41]; and interleukin 4, another proinflammatory cytokine implicated in the development of atopy, hypersensitivity, and Th2 T cell polarization, among other functions [42]. Lastly, several studies point to a role for apoptosis in AIH pathophysiology and treatment response.

### Emerging Therapies for AIH, With a Focus on Pathways

Regarding emerging therapies being studied in autoimmune hepatitis, the most prominent themes we identified in our literature search relate to the balance between T cell activation, which can lead to production of IL-2 and IL-17, and suppressive responses, namely, the function of Tregs. As anticipated, this corresponds directly to active studies targeting these pathways for treatment of AIH, with 4 clinical trials listed on ClinicalTrials.gov examining the role of IL-2, IL-17, and Treg biology in AIH. This is an active area of treatment research.

Regarding the role of apoptosis, there are two different ongoing clinical trials in AIH patients listed on ClinicalTrials.gov, one studying the immunoproteasome inhibitor, zetomipzomib, and another studying an inhibitor of the receptor for B cell activating factor belonging to the tumor necrosis factor family, also known as a BAFF. Treatment with these drugs induces apoptosis of plasma cells; studies are ongoing to further define their efficacy and positioning within the AIH treatment armamentarium.

Clinical trials were not listed on ClinicalTrials.gov for the remaining literature trends identified in Fig. 1. However, regarding IL-6, tocilizumab is a medication that blocks the IL-6 receptor, inhibiting IL-6 signaling; this medication is part of the standard of care for treatment of other autoimmune diseases such as rheumatoid arthritis (RA). Like AIH, RA is characterized by marked B cell activation, and numerous phase III clinical trials have demonstrated the efficacy of tocilizumab in RA [41]. This could be a future area of study to determine whether there is a role for tocilizumab in the treatment of AIH. While there are no ongoing trials of IL-4-related therapy in AIH, one could envision a future study, given the role of IL-4 in allergy and hypersensitivity [42]. Perhaps IL-4 could play a role in drug-induced autoimmune hepatitis, opening a window to develop more personalized approaches to the treatment of AIH subtypes.

## Conclusions

Therapy for AIH often needs to move beyond first-line agents to second- and third-line medications, like MMF and tacrolimus (Table 2). There remain multiple unmet needs in AIH, including explanations for gender-, race-, and ethnicity-based disparities, pathophysiologic processes, and targeted therapies. Our systematic review revealed Tregs, cytokines, and apoptosis as recurring themes in the literature. Major advances in the field will only come about through multicenter collaborations, as AIH is a relatively rare disease. These collaborations will hopefully lead to translational studies revealing mechanisms, upon which novel therapeutic strategies can be based.

## Supplementary Material

Supplementary1

**Supplementary Information** The online version contains supplementary material available at https://doi.org/10.1007/s11901–024-00657–4.

## Figures and Tables

**Table 1:** Overview of therapy options for second- and third-line use

Drug name	# of participants	Dosing	Adverse effects

Mycophenolate Mofetil	*n* = 22 (Giannakopoulos et al., 2019) [12] *n* = 50 (Kolev et al., 2022) [15] *n* = 18 (Liberal et al., 2021) [16] *n* = 105 (Roberts et al., 2018) [17]	Starting dose: dose of 20 mg/kg/day Max dose: 3 g/day Starting dose: 2 × 500 mg/day or 2 × 1 g/day Starting dose: 500 mg/day Max dose: 2000 mg/day Starting dose: 1 g/day Max dose: 2.0 g/day	Teratogenic Hematologic suppression Gastrointestinal side effects Headache Infection Increased risk of malignancy
	*n* = 121 (Efe et al., 2017) [20••]	0.5–2 g/day	
	Pediatrics: *n* = 18 (Efe, 2018) [19]	20–40 mg/kg twice a day	
Tacrolimus	*n* = 16 (Roberts et al., 2020) [21]	Starting dose: 2 mg/day Max dose: 4 mg/day	Neurological side effects Ototoxicity
	*n* = 23 (Ferre-Aracil et al., 2021) [22] *n* = 10 (Pape et al., 2020) [25]	Managed by trough levels - <5 ng/mL in 7 patients - ≥5 ng/mL in 15 patients Initial dose: 0.08 mg/kg in 2nd line and 0.06 mg/kg in 3rd line Max dose: 0.04 mg/kg in 2nd line and 0.07 mg/kg in 3rd line	Renal failure Diabetes Hypertension GI side effects Increased risk of malignancy Monitor with trough levels
	*n* = 80 (Efe et al., 2017) [20••]	1–8 mg/day	
	Pediatric: *n* = 20 (Efe et al., 2018) [19]	0.05–0.1 mg/kg twice daily Maintained trough level <6 ng/dL	
Cyclosporine	*n* = 10 (Pape et al., 2019)	Initial dose: 2.11 mg/kg as 2nd line and 1.83 mg/kg as 3rd line Max dose: 2.11 mg/kg for 2nd and 3rd line	Hypertension Neurological side effects GI symptoms
	*n* = 17 (Roberts et al., 2020) [21] Pediatric: *n* = 8 (Nastasio et al., 2019) [24]	Initial dose: 120 mg/day Max dose: 188 mg/day 1.5–8 mg/kg maintained via trough levels Initial trough levels: 150–200 ng/mL Once in remission, maintain trough levels 100–150 ng/mL, and then between 50 and 70 ng/mL after 1 year of treatment	Hematologic suppression Renal impairment Gingival hyperplasia Skin disorders Increased risk of malignancy
Sirolimus	*n* = 5 (Chatrath et al., 2014) [27]	Initial dose: 2 mg/day Increased until serum levels of 10–20 ng/dL	Increase in cholesterol and triglyceride levels
	Pediatric: *n* = 6 (Kerkar et al., 2005) [28]	Initial dose: 1–3 mg/day Titrated to maintain levels of 5–8 μg/dL	Infection
Infliximab	*n* = 11 (Weiler-Normann et al., 2013) [32]	5 mg/kg at time point 0, 2 weeks, 6 weeks, and then every 4–8-week pending response	Infection Hepatotoxicity Allergic reaction to infusion
Belimumab	*n* = 2 (Arvaniti et al., 2020) [37]	10 mg/kg	Headache
	*n* = (Kolev et al., 2023) [38]	200 mg once weekly	Burning while injection
Rituximab	*n* = 22 (Than et al., 2019) [36]	Two 1000-mg doses of rituximab 2 weeks apart 1 patient received 375 mg/m^2^	Infection
**Methotrexate**	*n* = 11 (Haridy et al., 2018) [29] *n* = 1 (Efe 2018) [19] *n* = 2 (Sultan 2011) [30]	7.5–20 mg/week 15 mg/week 10 mg/m^2^/week	Hepatotoxicity Pulmonary fibrosis Hematologic suppression Teratogenic

**Table 2:** Progression of therapy for different AIH phenotypes