# Supplementary material for: AIH Therapy: Beyond First‑Line
Source: Curr Hepatol Rep. Author manuscript; Available in PMC 2026 Jan 24. (PMC12830046; doi:10.1007/s11901-024-00657-4)
Supplement: Supplementary1 [file NIHMS2117103-supplement-Supplementary1.docx]

**Appendix 1.**

((((((("Hepatitis, Autoimmune"[Majr] OR “Autoimmune Hepatitides” OR “Autoimmune Chronic Hepatitis” OR "Autoimmune Hepatitis" OR “autoimmune chronic active hepatitis” OR “Autoimmune liver disease” OR “Autoimmune liver diseases” OR "immune mediated liver disease" OR "immune mediated liver diseases" OR (("AILD" OR "AIH" OR “immune tolerance”[mh] OR “break in tolerance”) AND (liver OR hepatic OR hepatocellular))))))

AND (((“flow cytometry”[mh] OR “flow cytometry” OR “microarray analysis”[mh] OR microarray OR RNA-seq OR “exome sequence” OR “exome sequences” OR “exome sequencing” OR “enzyme linked immunosorbent assay” OR “enzyme-linked immunosorbent assays” OR ELISA OR immunohistochemical OR immunohistochemistry OR “gene sequence” OR “gene sequences” OR “gene sequencing” OR PCR OR “polymerase chain reaction” OR (BLAST AND sequence*) OR “hierarchical cluster” OR “hematoxylin and eosin staining” OR “haematoxylin and eosin staining” OR genotyp* OR haplotype* OR immunophenotyp* OR “liver biopsy” OR “liver biopsies” OR “needle biopsy” OR immunoperoxidase)))))

AND ((DNA OR RNA OR gene OR genes OR cytokine OR cytokines OR lymphokine OR lymphokines OR chemokine OR chemokines OR interleukin OR interleukins OR IL-* OR cell OR “IL-“ OR signature OR signatures)))
